# Obstetric Surgery

**Published:** 1895-12

**Authors:** 


*Obstetric Surgery.* By EGBERT H. GRANDIN, M.D., and GEORGE W. JARMAN, M.D. Pp. ix., 207. Philadelphia: THE F. A. DAVIS COMPANY. London: F. J. REBMAN. 1895.

This work, by the Obstetric Surgeons to the New York Maternity Hospital, has many of the excellencies and not a few of the peculiarities of American medical publications.

The first part of *Obstetric Surgery* to engage our attention is its "Introduction," so-called, but which is in fact a rather long and elaborate description of Obstetric Asepsis and Antisepsis. We think that our American *confrères* exaggerate more than we do, in Europe, the all-importance of thorough-going asepsis. This, however, is a fault on the right side, and much better than the opposite extreme. In *Obstetric Surgery*, for instance, we find painfully minute directions for accomplishing the thorough antisepsis of the patient herself, such as savour very much of the *nimia Medici diligentia*, and to which we should have difficulty in inducing our countrywomen to submit. It is stated: "If the required manipulations are in the vagina, a new tooth-brush [!] should be inserted into the canal, and this should be also scrubbed with soap and water. It is next to be scrubbed with a solution of bichloride of mercury (1 to 1000)." The probable effect of the rough treatment thus described would be abrasion of epithelium, and then mercurial poisoning. Is it worth while to run this risk, when we are told subsequently: "We have endeavored to emphasize our belief, and this is the current belief, that the lying-in woman is septicized solely through personal contact. By this we mean that the atmosphere is not a factor, and that the infectious material does not originate in the body of the woman"? The following little paragragh, with its *couleur de rose* view of parturient perils, forms both a fitting close and climax to this aseptic "Introduction." "*Aseptic* and *elective* obstetrics rob labor of its terrors and the puerperal state of well-nigh its sole risk." But, how about hemorrhages, accidental, unavoidable, and *post-partum?*

Chapter I. treats of "Obstetric Dystocia and its determination." Before entering upon this chapter, we wish to say a few words as to the manner in which the publishers have done their work. It deserves unqualified approval. The paper and type are excellent and the illustrations unexceptionable; and the book on the whole is beautifully got up. As regards the illustrations, the majority are woodcuts and are borrowed from well-known standard works on Midwifery; for which service the authors duly express their obligations. There are altogether eighty-four woodcuts. We must, however, notice more in detail a prominent feature of the book—the fifteen full-page plates; these are from photographs, and "have been prepared from nature under the personal supervision of the authors." Now, these photographs are very well executed, and we have applied the term "unexceptionable" to them as well as to the woodcuts, but solely as works of art. On the score of good taste, however, they are decidedly *exceptionable.* For instance, in Plate III., a woman has been photographed lying on her back, with bare abdomen, thighs, nates, and external parts fully exposed, whilst a surgeon is digitally examining her, and a young man (probably a pupil) and a young nurse in No. 2 of Plate III. are holding her thighs in good position and in the most unconcerned manner. Now, we all know that medical men (or women) ought not to be squeamish about such proceedings, were it impossible to otherwise procure correct diagrams for teaching purposes—as the preface expresses it, for the "special end in view of teaching graphically." But the picture in question does not do this; it only shows the ordinary position on the back which in America and on the Continent is usually adopted during obstetric operations. To subject a patient to such an ordeal as this in order to achieve so small a result is, we venture to think, morally indefensible. If we could render the integuments and the viscera and their contents, of a woman during labour, as transparent as those of a rotifer under the microscope, photographs would soon supersede diagrams, but at present we must rest contented with the latter. The same objections on the ground of inutility, though not of course of indelicacy, apply to photographs of obstetric operations on the mannikin such as Plate IV. Here the hands of the operator holding the forceps are most life-like and natural, but no idea whatever is given of the relative position of the forceps, the head, and the pelvis. With the exceptions we have mentioned, Chapter I. is a very good and practical one, and also very complete and exhaustive, good qualities in which the Americans, especially in their larger works, much resemble the Germans. There are a number of descriptions and diagrams of instruments, chiefly for external pelvimetry, the first of which we recognise as the celebrated callipers of Baudelocque. (But why, throughout this book, is the first syllable of the great French accoucheur's name always spelt Beau—instead of Bau?) After all, these external pelvic measurements are not of much practical value, and we quite agree with the authors, that for determining the internal measurements, "the finger and, if need be, the hand" (under anæsthesia) "best subserve the purpose." There are also in this chapter some very good woodcuts of pelvic distortions of various kinds, and descriptions of diseased conditions and other causes which produce them.

In Chapter II. Artificial Abortion and the Induction of Premature Labour are treated, and the morbid conditions which would justify one alternative or the other are discussed with much good sense and judgment, especially in carefully balancing the often conflicting interests of mother and child. This is followed by a useful series of diagrams representing the various instruments suitable for either proceeding. We have had considerable experience of these operations, and on the whole recommend the instruments and the mode of inducing labour adopted by Dr. Barnes.

In Chapter III., which treats of the Forceps and which is one of the best in the book, there are some excellent woodcuts of various instruments of this kind. These are most useful and practical. Elliott's forceps, which seems to be in very general use in America, is, in our opinion, too short in the shanks. Barnes's long forceps, perhaps the best and most used in England, is not here represented. We observe that in certain exceptional cases of breech presentation, the authors recommend the application of the forceps to the breech in order to expedite delivery. In this respect we do not agree with them. If the forceps do not slip off, which is the most likely thing to happen, the extremities are almost sure to make most injurious pressure on large and important viscera, such as the liver, spleen, or heart, and thus most probably occasion the death of the child.

Had time and space permitted, we should gladly have dealt at some length on Chapter IV., for it is the longest, the ablest, and the best illustrated in the book, besides being perhaps the most interesting. In the next two chapters Symphysiotomy and the Cæsarean Section are considered. The concluding chapters deal with Embryotomy and the Surgery of the Puerperium.

This work, written from the surgical point of view of obstetrics, is one which would be read with great advantage by rising accoucheurs in general, but *par excellence* by those who aspire to the important post of obstetric surgeon to a hospital.

